# Infusing Sodium Bicarbonate Suppresses Hydrogen Peroxide Accumulation and Superoxide Dismutase Activity in Hypoxic-Reoxygenated Newborn Piglets

**DOI:** 10.1371/journal.pone.0039081

**Published:** 2012-06-22

**Authors:** Jiang-Qin Liu, Namdar Manouchehri, Tze-Fun Lee, Mingzhu Yao, David L. Bigam, Po-Yin Cheung

**Affiliations:** 1 Department of Neonatology, Shanghai First Maternity and Infant Hospital, Tongji University School of Medicine, Shanghai, People’s Republic of China; 2 Department of Pediatrics, University of Alberta, Edmonton, Alberta, Canada; 3 Department of Surgery, University of Alberta, Edmonton, Alberta, Canada; Hôpital Robert Debré, France

## Abstract

**Background:**

The effectiveness of sodium bicarbonate (SB) has recently been questioned although it is often used to correct metabolic acidosis of neonates. The aim of the present study was to examine its effect on hemodynamic changes and hydrogen peroxide (H_2_O_2_) generation in the resuscitation of hypoxic newborn animals with severe acidosis.

**Methods:**

Newborn piglets were block-randomized into a sham-operated control group without hypoxia (n = 6) and two hypoxia-reoxygenation groups (2 h normocapnic alveolar hypoxia followed by 4 h room-air reoxygenation, n = 8/group). At 10 min after reoxygenation, piglets were given either i.v. SB (2 mEq/kg), or saline (hypoxia-reoxygenation controls) in a blinded, randomized fashion. Hemodynamic data and blood gas were collected at specific time points and cerebral cortical H_2_O_2_ production was continuously monitored throughout experimental period. Plasma superoxide dismutase and catalase and brain tissue glutathione, superoxide dismutase, catalase, nitrotyrosine and lactate levels were assayed.

**Results:**

Two hours of normocapnic alveolar hypoxia caused cardiogenic shock with metabolic acidosis (pH: 6.99±0.07, HCO_3_^−^: 8.5±1.6 mmol/L). Upon resuscitation, systemic hemodynamics immediately recovered and then gradually deteriorated with normalization of acid-base imbalance over 4 h of reoxygenation. SB administration significantly enhanced the recovery of both pH and HCO_3−_ recovery within the first hour of reoxygenation but did not cause any significant effect in the acid-base at 4 h of reoxygenation and the temporal hemodynamic changes. SB administration significantly suppressed the increase in H_2_O_2_ accumulation in the brain with inhibition of superoxide dismutase, but not catalase, activity during hypoxia-reoxygenation as compared to those of saline-treated controls.

**Conclusions:**

Despite enhancing the normalization of acid-base imbalance, SB administration during resuscitation did not provide any beneficial effects on hemodynamic recovery in asphyxiated newborn piglets. SB treatment also reduced the H_2_O_2_ accumulation in the cerebral cortex without significant effects on oxidative stress markers presumably by suppressing superoxide dismutase but not catalase activity.

## Introduction

Sodium bicarbonate (SB) has been used by many clinical practitioners to treat metabolic acidosis of asphyxiated neonates during and after resuscitation. Recently, evidence indicated that SB therapy may be useless [1], [2], [3]. Although the effect of SB on long-term neurodevelopmental outcome has not been assessed, administrating SB during resuscitation not only does not improve the survival or immediate neurologic outcome [4], but also increases risk of death or intraventricular hemorrhage [5]. Indeed, it may be dangerous to use SB during hypoxia-ischemia of neonates because of paradoxical acidosis [2], [6], [7]. Interestingly, in a recent questionnaire survey, over 40% of consultant neonatologists in Europe would prescribe SB to asphyxiated neonates with severe metabolic acidosis [8]. Therefore, the effectiveness of SB in the overall recovery of this particular group of infants needs to be further examined.

Asphyxiated neonates commonly develop cardiovascular dysfunction after resuscitation including hypotension, cardiogenic shock, pulmonary hypertension and cerebral hypoperfusion or hypoxic-ischemic encephalopathy. The etiology of the brain damage is likely to be multifactorial, however, there is growing evidence that free radicals are important for the reoxygenation and/or reperfusion injury following asphyxia [9], [10], [11]. In addition to free radicals, reactive oxygen species (ROS) are generated including the superoxide anion, which is converted to hydrogen peroxide (H_2_O_2_) by superoxide dismutase (SOD). Excess production of superoxide anions may react with H_2_O_2_ producing toxic hydroxyl radicals in the presence of ferrous form of iron. The hydroxyl radicals can cause cellular damage and apoptotic cell death by oxidizing proteins, inducing lipid peroxidation and damaging DNA. Hypoxic-ischemic injury has been shown to inhibit the activity of SOD, a key enzyme of converting superoxide to H_2_O_2_ [12]. Similar to this observation, an increase in plasma superoxide, but not H_2_O_2_, has been reported in patients with bicarbonate hemodialysis [13]. Conversely, a rapid increase in H_2_O_2_ production has been shown in guard cells incubated with 2 mM bicarbonate [14]. Thus far, no study has been carried out to examine effect of SB on ROS generation in animals after either ischemia-reperfusion or hypoxia-reoxygenation. Using an acute swine model of neonatal asphyxia, we investigated the effects of SB on hemodynamic recovery and H_2_O_2_ generation after hypoxia-reoxygenation. We hypothesized that administering SB in the resuscitation of asphyxiated newborn piglets would have no systemic hemodynamic benefits but reduce the production of H_2_O_2_. We also examined secondary outcomes including pulmonary and carotid hemodynamics, enzymes and metabolites related to H_2_O_2_ production including SOD and catalase, nitrotyrosine (a marker of peroxynitrite production) and glutathione redox (oxidized glutathione (GSSG)/total glutathione (GSH)) ratio, a marker of oxidative stress [15], [16], [17].

## Methods

All experiments were conducted in accordance with the guideline and approval of the Animal Care and Use Committee, University of Alberta. Both genders of 1–4 days old newborn Yorkshire-Landrace piglets, weighing 1.6 to 2.5 kg, were used.

### Animal Preparation

The animal preparation was similar to that described previously with minor modifications [18], [19]. Briefly, anesthesia was initially maintained with inhaled isoflurane (2–3%), which was then switched to fentanyl (0.005–0.05 mg/kg/h), midazolam (0.1–0.2 mg/kg/h) and pancuronium (0.05–0.1 mg/kg/h) once mechanical ventilation was established. Oxygen saturation was continuously monitored with a pulse oximeter (Nellcor, Hayward, California). Heart rate and blood pressure were measured with a 78833B monitor (Hewlett Packard Co, Palo Alto, California). Fractional inspired oxygen concentration (FiO_2_) was measured by an oxygen monitor (Catalyst Research, Owings Mills, MD) and maintained at 0.21–0.24 for oxygen saturation between 90 and 97%. Maintenance fluids during experimentation consisted of 5% dextrose at 10 ml/kg/h and 0.9% normal saline solution at 2 ml/kg/h. The body temperature was maintained at 38.5–39.5°C using an overhead warmer and a heating pad.

### Surgical Instrumentation

Under anesthesia, piglets were catheterized with Argyle catheters (5F: Sherwood Medical Co., St. Louis, MO) via the right femoral artery and vein to the level of infra-renal aorta and right atrium, respectively. The arterial catheter was used to monitor mean arterial pressure (MAP), whereas venous catheter was used for monitoring central venous pressure with a 78833B monitor (Hewlett Packard) and administrating fluids and medication. After tracheotomy was established through a midline neck incision, the left common carotid artery was isolated and circled by a 2-mm transit time ultrasound flow probe (2SS, Transonic Systems Inc., Ithica, NY) for measuring common carotid blood (CCA) flow. A left anterior thoracotomy was performed to expose the main pulmonary artery. A 6 mm transit time ultrasound flow probe (6SB, Transonic Systems Inc., Ithaca, NY) was placed around the main pulmonary artery to measure pulmonary blood flow. A 20G angio catheter (Insyte-W, Becton Dickinson Infusion Therapy Systems Inc., Sandy, Utah) was inserted for the measurement of pulmonary artery pressure (PAP).

The piglet was then placed in a prone position with the head mounted in a stereotaxic holder. Using bregma as the reference point, a stainless steel guide cannula (19-gauge) was implanted (at a depth of 6 mm) in the right frontoparietal cortex using the following co-ordinate: antero-posterior = 6.5 and lateral = 4 mm. The piglet was replaced in the right-sided position after cannula implantation. All incisions were closed to minimize evaporative heat loss. After all surgical procedures were completed, pancuronium infusion was discontinued.

The incision was covered to minimize evaporative heat loss. Heart rate, MAP, PAP, pulmonary artery and CCA flows were continuously monitored and all data were recorded by a DT 2801-A analogue to digital converter board (Data Translation, Ontario, Canada). For each hemodynamic parameter, an average of a two-minute range at specific timepoints during the experimental period was taken. Cardiac index, a surrogate estimated by the pulmonary artery flow, and CCA flow index were corrected for individual piglet mass. Oxygen delivery in CCA was calculated by CCAFI × arterial O_2_ saturation × hemoglobin concentration×1.34+ arterial O_2_ partial pressure ×0.003.

### Experimental Protocol

**Figure 1:**
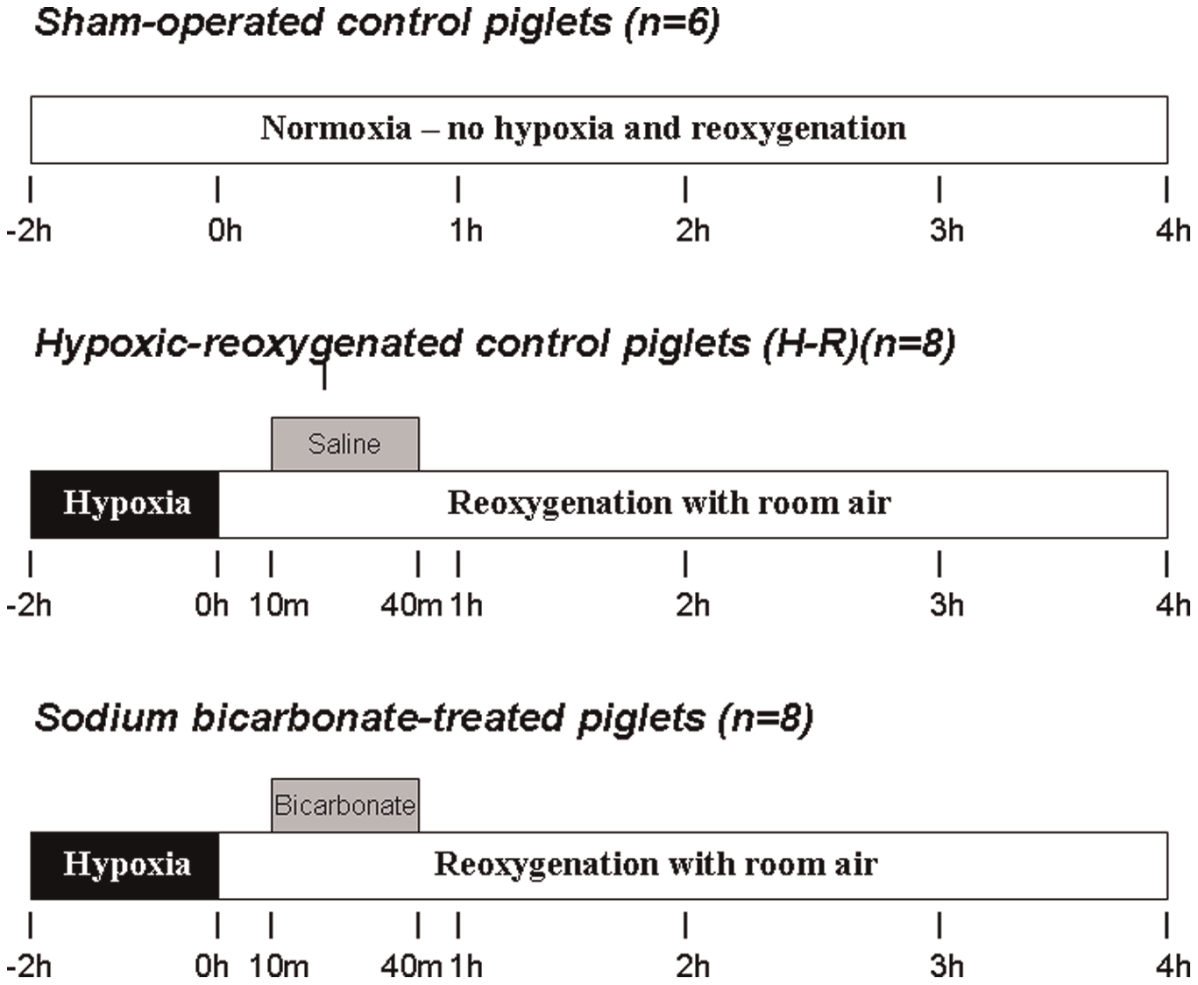
Experimental groups and protocol.

After surgery, animals were stabilized for at least 60 min. As shown in Fig 1, piglets were divided into a sham-operated control group without hypoxia-reoxygenation throughout the experimental period (n = 6) and two hypoxia-reoxygenation experimental groups. The 2 h of normocapnic alveolar hypoxia was induced by decreasing the FiO_2_ of 0.11–0.15 using nitrogen and oxygen gas mixture to achieve severe metabolic acidosis and hypoxemia (pH∼7.0, HCO_3_^−^∼10 mmol/L, PaO_2_ 20–40 mmHg). After hypoxia, hypoxia-reoxygenation piglets were reoxygenated with room-air and block-randomized into hypoxia-reoxygenation control or SB-treated groups (n = 8/group). Ten minutes after reoxygenation, piglets received intravenously either 4.2% SB (2 mEq/kg, 1∶1 v/v diluted with sterile water) or saline (equal volume)(hypoxia-reoxygenation control) infusion over 30 min in a blinded manner. The dosage was based on the clinical guideline [20]. During the administration of SB, peak inspiratory pressure (18–25 cm H_2_O) and respiratory rate (12–20 breaths/min) were adjusted to maintain normocapnia (35–45 mmHg minimizing the effect on pH). At the end of the experiment, the piglet was euthanized with an overdose of pentobarbital (100 mg/kg, i.v.). After removing rapidly from the skull, the brain was flash-frozen in ice-cold isopentane and then stored at –80°C for subsequent analysis.

### Cerebral Cortical Hydrogen Peroxide Measurement

Changes in cortical H_2_O_2_ during hypoxia-reoxygenation were measured directly by electrochemical H_2_O_2_ sensor (HPO-100, World Precision Instruments Ltd. Sarasota, FL). The H_2_O_2_ sensor was inserted through the guide cannula into the cortex area. The sensors were connected to a computer-controlled data acquisition system (Apollo 4000, World Precision Instruments Ltd.). The signal outputs were recorded continuously throughout the experiment. Immediately before and after each experiment, the H_2_O_2_ sensor was calibrated with 1 mM H_2_O_2_ in phosphate buffer (10 mM, pH 7.4) according to manufacturer’s instructions. The mean value was used for converting the signal outputs. The relative changes in cortical H_2_O_2_, expressed in µM, were calculated with reference to the normoxic baseline after stabilization.

### Biochemical Analysis

A block of cortical tissue (5×5×5 mm^3^) from the left side of the cortex corresponding to the cannulation area of the right side was dissected. Following the manufacturer’s suggested protocol in the assay kits, tissues were homogenized with 10 µl/mg of 50 mM phosphate buffer containing 1 mM EDTA (pH 7.0). The levels or activities of GSSG and GSH, SOD, catalase and nitrotyrosine were measured using commercially available assay kits (Cayman Chemical, #703002, #706002 and #707002, respectively).

Total GSH (GSH+GSSG) content was quantified by using glutathione reductase to convert GSSG to GSH. The sulfhydryl group of GSH reacted with 5.5′-dithiobis-2-nitrobenzoic acid to produce yellow colored 5-thio-2-nitrobenzoic acid which was then measured colorimetrically at 405 nm. The amount of GSSG was measured after derivatizing GSH with 2-vinylpyridine. The glutathione redox (GSSG/total GSH) ratio was calculated and used as an indicator of oxidative stress as described by others [15], [16], [17].

The activities of plasma SOD (extracellular SOD) and brain tissue total SOD (extracellular, cytosolic and mitochondrial SOD) were measured by utilizing tetrazolium salt to detect superoxide radicals generated by xanthine oxidase and hypoxanthine. One unit of SOD was defined as the amount of enzyme required to exhibit 50% dismutation of superoxide radicals.

The catalase assay kit was based on the reaction of catalase with methanol in the presence of H_2_O_2_ and the formaldehyde produced was then measured colorimetrically with Purpald as the chromogen.

The content of nitrotyrosine in brain cortex was measured using an ELISA kit (Cell Biolabs, #STA-305). Tissue lactate was assayed by enzymatic spectrometric methods. The protein content was determined by bicinchoninic acid assay kit (Sigma).

### Statistical Analysis

All results were expressed as mean±SD. One-way and two-way repeated measures analysis of variance (ANOVA) tests were used to study the differences among groups as appropriate. *Post-hoc* testing with Student-Newman-Keuls method was performed for pairwise comparisons with the hypoxia-reoxygenation control group. Statistical analyses were performed using SigmaStat® (V. 2.0, Jandel Co.). Significance was set at p<0.05.

## Results

There were no differences in body weight and age among three groups (weight: 1.9±0.3, 1.9±0.2 and 2.1±0.3 [kg]; age: 3.3±1.0, 2.7±0.7 and 3.1±1.1 [day] for sham-operated control, hypoxia-reoxygenation control and SB-treated groups, respectively). There were no statistical differences in hemodynamic parameters and blood gas among three groups at the baseline (Tables 1 and 2).

### Effect of SB Treatment on Arterial Blood Gas and Acid-base Status

Severe metabolic acidosis (mean pH: 6.99±0.07, mean HCO_3_^−^: 8.54±1.63 mmol/L) developed after 2 h of normocapnic alveolar hypoxia (Table 1). Upon reoxygenation, pH and HCO_3_^−^of hypoxia-reoxygenation control piglets recovered gradually towards the respective normoxic baseline values, but remained significantly lower than those of sham-operated control piglets at the end of experiment. Treating the animals with SB, the recovery of both pH and bicarbonate levels were significantly faster during the first hour of reoxygenation with higher levels throughout the experimental period when compared with those of hypoxia-reoxygenation controls (Table 1). Plasma lactate increased at 2 h of hypoxia in two hypoxia-reoxygenation groups and declined gradually back to their respective baseline values after reoxygenation with no significant effect noted in the SB-treated group (Table 1).

**Table 1:** Changes in arterial blood gas during hypoxia and reoxygenation.

	Normoxic Baseline	2h of hypoxia		Reoxygenation with room air (minutes)					
				10	25	40	60	120	240
**pH**									
Sham-operated controls†	7.40±0.03		7.42±0.03*	7.43±0.04*	7.44±0.03*	7.44±0.04*	7.43±0.05*	7.43±0.04*	7.43±0.06*
Hypoxic-reoxygenated controls	7.42±0.06		6.98±0.08	6.95±0.10	7.07±0.07	7.14±0.06	7.20±0.06	7.33±0.06	7.32±0.06
Bicarbonate-treated group	7.40±0.03		7.00±0.06	6.98±0.10	7.13±0.08*	7.23±0.06*	7.28±0.07*	7.38±0.06	7.37±0.05
**PaO_2_ (mmHg)**									
Sham-operated controls	78±23	69±6*		67±4	68±3	68±7	58±9	66±3	64±3
Hypoxic-reoxygenated controls	78±20	35±6		76±9	82±17	76±17	67±7	67±8	67±11
Bicarbonate-treated group	68±11	35±7		79±20	78±12	81±18	74±19	68±8	66±8
**PaCO_2_ (mmHg)**									
Sham-operated controls	37±3	39±1		37±3*	40±4	37±2	37±5	38±4	40±5
Hypoxic-reoxygenated controls	38±5	40±5		45±5	40±3	40±4	42±2	40±2	43±3
Bicarbonate-treated group	41±3	43±5		47±9	41±5	41±5	41±2	40±3	44±3
**HCO_3_− (mmol/L)**									
Sham-operated controls†	23.3±2.1	25.6±1.7*		26.0±1.7*	26.4±2.9*	25.3±2.7*	25.0±2.7*	25.9±2.6*	26.0±2.7*
Hypoxic-reoxygenated controls	24.6±1.9	8.2±1.6		8.6±1.8	10.9±1.8	12.9±2.0	15.3±2.2	20.8±2.7	21.2±3.4
Bicarbonate-treated group	24.8±1.7	8.9±1.7		9.4±1.9	12.9±2.3	16.4±2.4*	18.7±3.0*	22.9±2.6	24.5±2.9*
**Lactate (mmol/L)**									
Sham-operated controls†	3.9±1.0	2.4±0.9*		2.1±0.8*	2.1±1.0*	2.0±0.9*	2.3±1.0*	1.8±0.7*	1.6±0.6*
Hypoxic-reoxygenated controls	4.4±0.9	18.3±2.5		17.1±2.6	14.9±2.4	12.7±2.3	10.7±2.8	5.8±2.6	4.0±3.1
Bicarbonate-treated group	3.2±0.7	16.2±2.5		15.2±2.5	13.2±2.6	10.7±2.9	8.6±2.4*	4.2±1.4	2.2±1.3

* p<0.05 vs. hypoxic-reoxygenated control group at the corresponding time point (two-way repeated measures ANOVA).

† p<0.05 vs. hypoxic-reoxygenated control group (two-way repeated measures ANOVA).

### Effects of SB on Systemic, Pulmonary and Carotid Hemodynamics

After 2 h of hypoxia, piglets had cardiogenic shock with hypotension and reduced cardiac index (Table 2). Upon resuscitation with room-air, MAP and cardiac index dramatically improved but then gradually decreased to values significantly below respective baseline and sham-operated control piglets after 40 min and 2 h of reoxygenation, respectively. Pulmonary hypertension gradually resolved with normalization of PAP. Despite slight tachycardia during the reoxygenation period, two hypoxia-reoxygenation groups did not differ regarding heart rate, MAP, PAP and cardiac index during the reoxygenation period, indicating that SB administration had no significant effects on systemic and pulmonary hemodynamics (Table 2).

**Table 2:** Changes in heart rate, mean arterial pressure and cardiac index during hypoxia and reoxygenation.

	Normoxic baseline		2h ofhypoxia	Reoxygenation with room air (minutes)							
				10			25	40	60	120	240
**Heart rate (beat per minute)**											
Sham-operated controls	206±8		205±33	202±29			204±26	208±26	206±34	200±32	201±28*
Hypoxic-reoxygenated controls	170±19		200±18	203±35			207±19	217±26	218±28	221±23	232±52
Bicarbonate-treated group	175±24		199±20	225±30			223±25	216±26	211±25	210±31	232±57
**Mean arterial pressure (mmHg)**											
Sham-operated controls †	66±8		59±7*		57±6		57±8	58±8*	58±7*	56±9	53±8*
Hypoxic-reoxygenated controls	83±15		30±3		63±20		49±10	46±8	47±7	49±8	42±10
Bicarbonate-treated group	81±10		28±2		59±10		48±6	43±5	42±4	48±5	44±7
**Pulmonary arterial pressure (mmHg)**											
Sham-operated controls		25±3	25±2			26±3	26±3	27±2	28±1	26±4	24±3
Hypoxic-reoxygenated controls		26±4	32±4			38±5	33±4	32±4	30±3	27±3	29±4
Bicarbonate-treated group		27±5	31±5			39±7	34±6	31±5	29±5	29±7	29±5
**Cardiac index (ml/kg)**											
Sham-operated controls	198±40		197±34*		194±31		199±31	202±28	203±40	194±39*	188±24*
Hypoxic-reoxygenated controls	205±58		91±27		180±57		152±73	172±65	171±52	148±43	128±56
Bicarbonate-treated group	188±27		94±43		192±49		166±27	142±19	138±25	144±29	135±48

* p<0.05 vs. hypoxic-reoxygenated control group at the corresponding time point (two-way repeated measures ANOVA).

† p<0.05 vs. hypoxic-reoxygenated control group (two-way repeated measures ANOVA).

**Figure 2:**
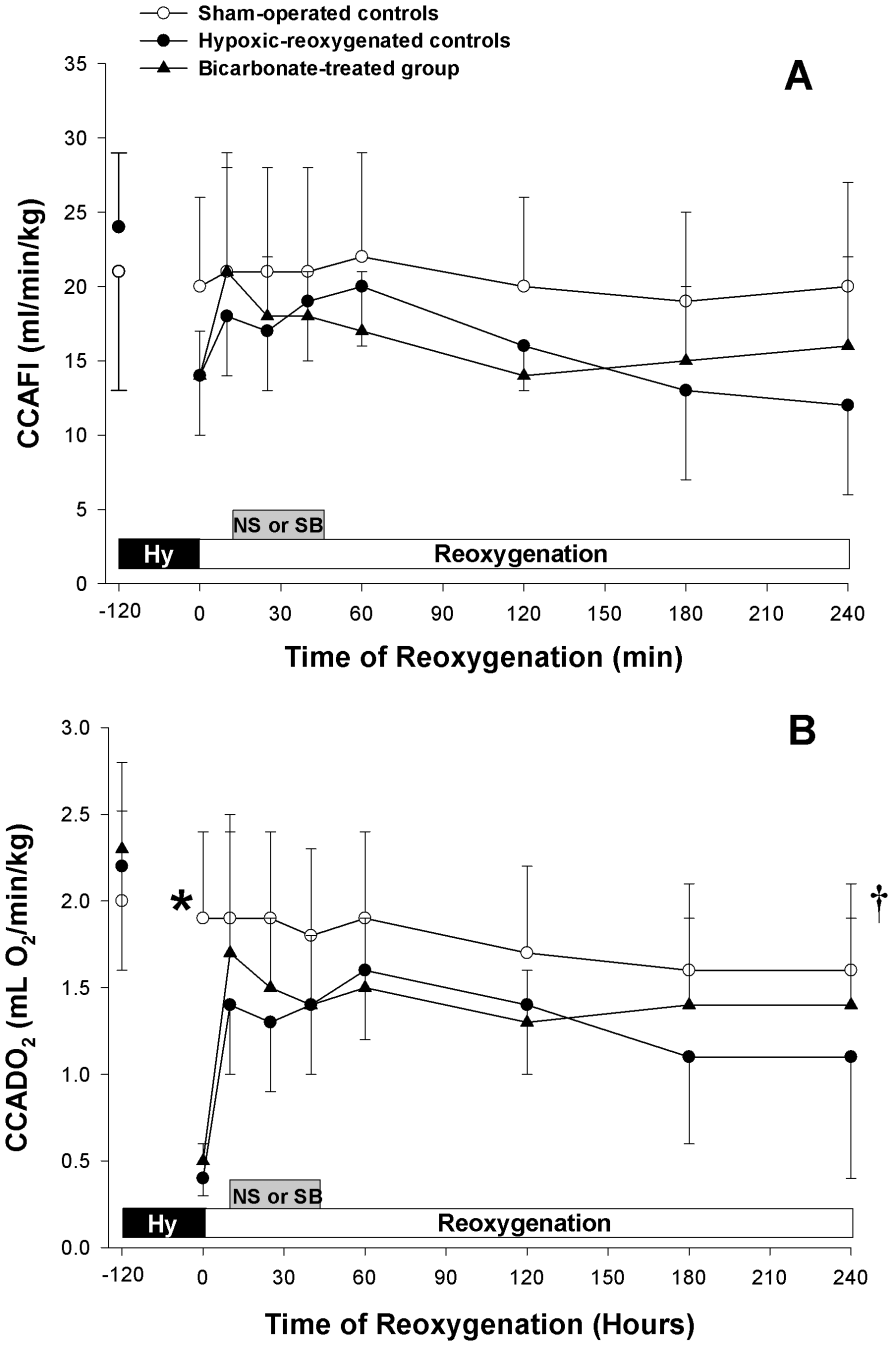
Common carotid artery perfusion. Temporal changes in (A) common carotid artery flow (CCAFI) and (B) common carotid arterial oxygen delivery (CCADO_2_) in sham-operated control piglets (○, Sham-operated controls, without hypoxia [Hy] and reoxygenation), hypoxia piglets receiving either saline (NS) (•, Hypoxic-reoxygenated controls) or sodium bicarbonate (SB) (▴, Bicarbonate-treated group) at 10 min after reoxygenation with room air. *p<0.05 vs. hypoxic-reoxygenated control group at corresponding time point, ^†^p<0.05 vs. hypoxic-reoxygenated controls during reoxygenation period (2-way repeated measures ANOVA).

As demonstrated in Fig 2A, CCA flow index significantly decreased in hypoxia-reoxygenation groups at 2 h of hypoxia (p<0.05). CCA flow index rose and was not different from that of sham-operated control group. There was no effect of SB infusion on CCA flow index during the recovery (Fig 2A). Corresponding to the changes of blood flow, the CCA oxygen delivery of hypoxia-reoxygenation control group decreased and remained significantly lower than that of the sham-operated control group during the recovery (Fig 2B).

### Effects of SB on Cerebral H_2_O_2_ Accumulation

**Figure 3:**
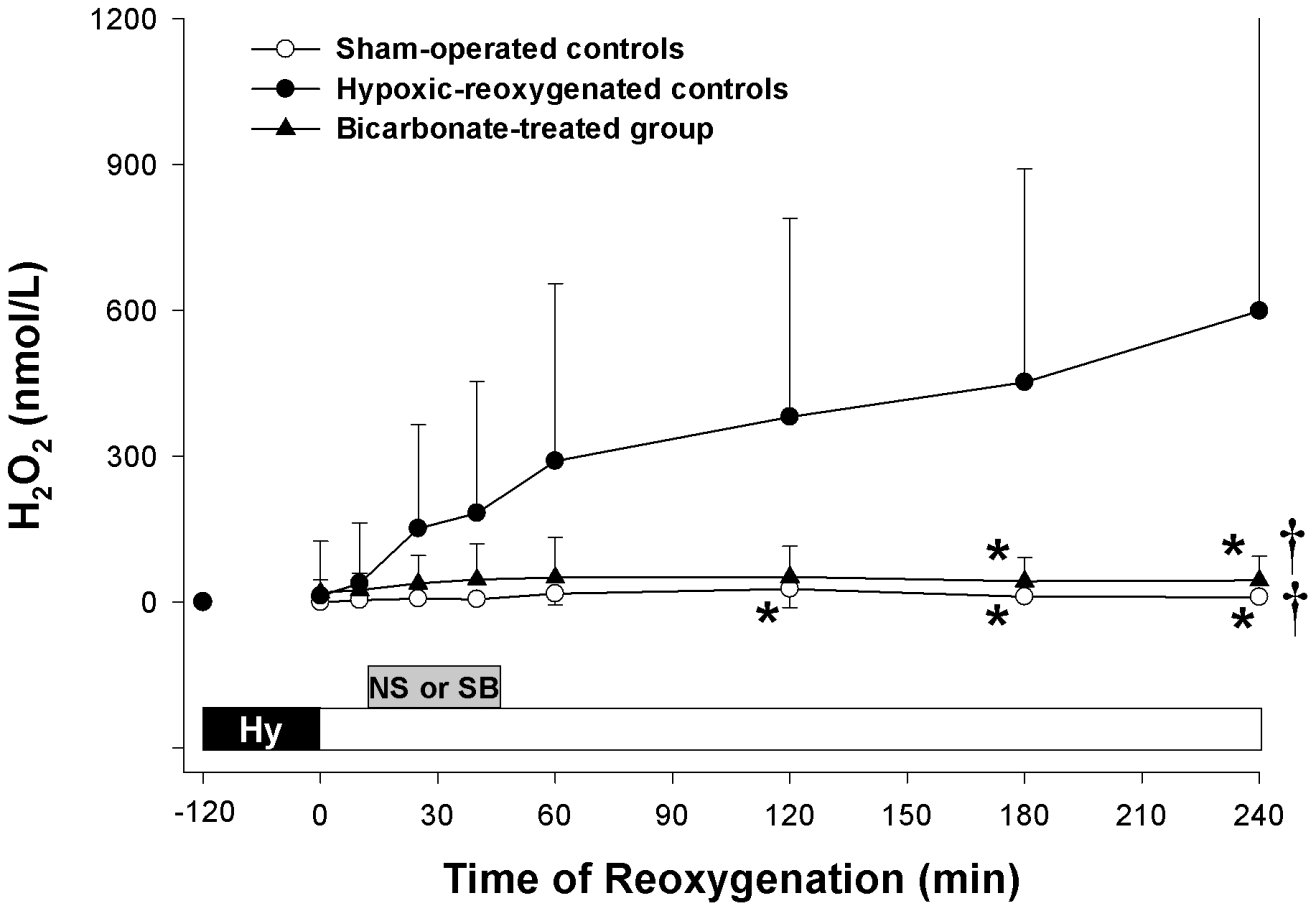
Cerebral hydrogen peroxide (H_2_O_2_) concentration. Temporal changes in H_2_O_2_ in the cerebral cortex of sham-operated control piglets (○, Sham-operated controls, without hypoxia [Hy] and reoxygenation), hypoxia piglets receiving either saline (NS) (•, Hypoxic-reoxygenated controls) or sodium bicarbonate (SB) (▴, Bicarbonate-treated group) at 10 min after reoxygenation with room air. *p<0.05 vs. hypoxic-reoxygenated control group at corresponding time point, ^†^p<0.05 vs. hypoxic-reoxygenated controls during reoxygenation period (2-way repeated measures ANOVA).

The temporal changes of cerebral H_2_O_2_ at the cortex during hypoxia-reoxygenation were shown in Fig 3. The level of cerebral H_2_O_2_ in the right cortex of hypoxia-reoxygenation controls increased steadily after reoxygenation and maintained at a high level throughout the remaining period. Treating the piglets with SB significantly suppressed the accumulation of H_2_O_2_ which was similar to the levels of sham-operated control piglets during reoxygenation (Fig 3).

### Effects of SB on SOD, Catalase, Nitrotyrosine and Glutathione Content

**Figure 4:**
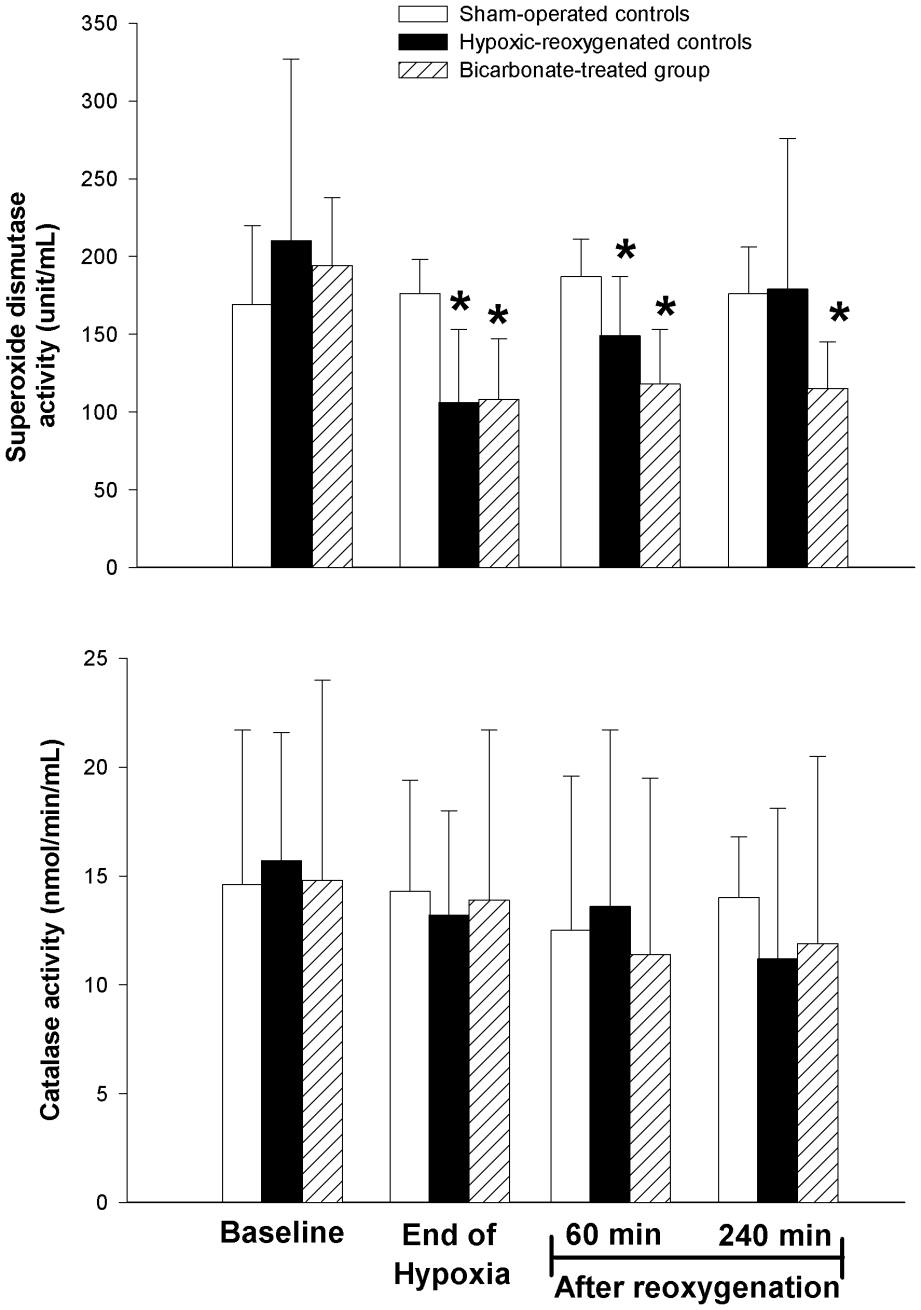
Plasma superoxide dismutase and catalase activities. Temporal activities of (A) superoxide dismutase and (B) catalase in the plasma of sham-operated control piglets (open bar, Sham-operated controls, without hypoxia and reoxygenation), hypoxia piglets receiving either saline (NS) (closed bar, Hypoxic-reoxygenated controls) or sodium bicarbonate (SB) (shaded bar, Bicarbonate-treated group) at 10 min after reoxygenation with room air. *p<0.05 vs. respective baseline at normoxia (2-way repeated measures ANOVA).

In the hypoxia-reoxygenation control group, the activity of plasma soluble SOD significantly decreased (about 50% of baseline) at the end of hypoxia and then recovered gradually during the reoxygenation period (Fig 4A). In contrast, the plasma SOD activity remained significantly lower than the baseline value during recovery in piglets treated with SB. However, there is no statistical significant difference among three experimental groups at the end of experimental period (Fig 4A). In contrast, the plasma activity of catalase remained steadily throughout the hypoxia-reoxygenation (Fig 4B). Although the cerebral SOD activity of SB-treated piglets was lower than that of both hypoxia-reoxygenation and sham-operated groups controls (Table 3), the differences were not significant. No differences in cerebral catalase activity were found among groups (Table 3). After hypoxia-reoxygenation, in the left cerebral cortex, tissue glutathione content, glutathione redox (GSSG/total GSH) ratio and nitrotyrosine levels were significantly different from those of sham-operated control group at the end of experiment (Table 3). Treating piglets with SB did not affect the cortical levels of these biochemical markers.

**Table 3:** Effects of bicarbonate on total glutathione (GSH), oxidized glutathione (GSSG), nitrotyrosine and lactate levels as well as superoxide dismutase (SOD) and catalase activities in cerebral cortex after hypoxic-reoxygenation.

Group	Group			p
	Sham-operated controls (n = 6)	Hypoxic-reoxygenated controls (n = 8)	Bicarbonate-treatedgroup (n = 8)	
Total GSH (µmol/mg protein)	252±13*	158±20	146±11	0.001
GSSG (µmol/mg protein)	25±2	27±3	18±3	0.12
GSSG/total GSH ratio	0.10±0.01*	0.18±0.01	0.13±0.02	0.02
Nitrotyrosine (nmol/mg protein)	37±7*	93±22	62±9	0.05
Lactate (µmol/mg protein)	1.81±0.70	2.32±0.45	1.79±0.55	0.11
SOD (units/mg protein)	196±31	209±30	173±37	0.12
Catalase (nmol/min/mg protein)	1.18±0.60	0.72±0.42	0.97±0.32	0.18

* p<0.05 vs. hypoxic-reoxygenated control group (one-way ANOVA).

## Discussion

In the present study, as expected, the administration of SB improved the recovery of pH and HCO_3_^−^ faster than those of hypoxia-reoxygenation controls, particularly during the first hour of reoxygenation. There was no significant effect of SB on systemic, pulmonary and carotid hemodynamics observed during hypoxia-reoxygenation. Treating the newborn piglets with SB also significantly reduced the level of H_2_O_2_ in the cerebral cortex, which was associated with the inhibition of SOD, but not catalase, activity.

Although the administration of SB in resuscitating the asphyxiated neonate is still debatable, our findings showed that the faster recovery in acid-base balance in piglets despite the ventilator adjustment to maintain normocapnia. It is important to note a poor correlation between blood gases and intracellular metabolic status, in addition to inadequately functioning of ATP-dependent ion pumps. Thus the blood gases and the related parameters should be very cautiously interpreted. Laptook [21] also previously reported that blood gas variables recovered much faster in hypoxic piglets treated with SB. Despite faster recovery on both pH and HCO_3_^−^, SB has no effect on systemic and pulmonary hemodynamic changes when compared with hypoxia-reoxygenation controls. Although the replenishment of base deficit with SB is supposed to have beneficial effects on myocardial contractility and organ perfusion, Cooper et al [22] suggested that extra SB may be detrimental to the myocardial cells as CO_2_ generating from SB diffused into the cells faster than HCO_3_^−^ (*paradoxical acidosis*). Furthermore, SB has also been shown to impair the effect of hypercapnia on increased cardiac output and carotid blood flow in an animal model of hypoventilation [23]. Our experimental design incorporates the ventilator adjustment that most practitioners do. This maneuver might have attenuated the acid-base alteration and associated paradoxical acidosis, which could lessen hemodynamic changes.

On the other hand, contradictory results have also been reported on the effect of SB on cerebral blood flow in various models of hypoxia/ischemia. Treating the rabbits with SB before induction of cerebral ischemia has been shown to improve the regional cortex blood flows [24]. Using near infrared spectrophotometry to detect the changes of oxyhemoglobin and deoxyhemoglobin, increased cerebral blood volume, but not blood flow velocity and oxygenation, has been reported after SB treatment in preterm infants [25]. Conversely, infusion of SB has been reported to either reduce the cerebral blood flow of asphyxiated newborns [26] and anesthetized dogs with global ischemia [27] or to have no effect on cerebral blood flow and oxygen delivery in hypoxic animals [21]. We did not show any effect on CCA blood flow and oxygen delivery in asphyxiated piglets treated with SB immediately after reoxygenation. Taken together, the administration of SB in the resuscitation of asphyxiated neonates at least is not beneficial to the cerebral blood flow and oxygen delivery to the brain.

To the best of our knowledge, it is the first study to report the effect of SB on ROS generation in the cerebral cortex of asphyxiated newborn subjects. Similar to our previous report [19], cortical H_2_O_2_ concentrations maintained around the baseline value during hypoxia, then increased gradually after resuscitation with room-air and became markedly elevated at 1 h after reoxygenation. This was associated with an increased oxidative stress, as indicated by higher glutathione redox ratio, in the cerebral cortex after hypoxia-reoxygenation. Post-resuscitation SB administration significantly attenuated the rise in H_2_O_2_ which remained at levels similar to that observed in sham-operated control piglets. It is well known that H_2_O_2_ is formed as a consequence of enzymatic dismutation of superoxide by SOD and its level is maintained by catalase and glutathione peroxides (GPX) [28]. Jung et al [12] previously reported that the expression of SOD decreased significantly during reperfusion in mice after cerebral ischemia. Similarly, a decrease in SOD activity in cultured hippocampal neurons was observed after hypoxia [29]. In the present study, the plasma SOD activity of asphyxiated piglets was markedly reduced after hypoxia and gradually recovered back to the baseline value by the end of experiment. Treating the piglets with SB abolished the recovery of SOD, of which activities remained low throughout reoxygenation. This in part supports the modestly decreased activity of SOD in the brain tissue of SB-treated piglets when compared to hypoxia-reoxygenation controls. The plasma and cortical catalase activities did not change with hypoxia-reoxygenation or SB administration. Although the activity of GPX was not measured directly in the present study, there was no significant effect of SB administration on cortical GSH content and GSSG/total GSH ratio. No change in brain catalase and GPX has also reported recently after HI in hippocampus and cortex of rat pups after hypoxia ischemia by carotid ligation [30]. Therefore, it is likely that SB suppressed H_2_O_2_ accumulation during hypoxia-reoxygenation by inhibiting the dismutation effect of SOD, rather than the metabolic clearance by catalase and GPX.

In biological systems, superoxide also reacts with nitric oxide to form peroxynitrite anion (ONOO^−^), a substance that has been implicated as a culprit in the pathogenesis of many diseases including hypoxia-reoxygenation [31], [32]. We here observed increased nitrotyrosine levels in the brain tissue of hypoxia-reoxygenation piglets. Theoretically, an increase in superoxide bioavailability by inhibiting the activity of SOD after SB administration should result in more ONOO^−^ formation, and thus its marker nitrotyrosine. Surprisingly, the cortical level of nitrotyrosine of SB-treated piglets was not affected. Although nitrotyrosine is derived from the oxidation of phenylalanine caused by peroxynitrite [33], the surrogate measurement of nitrotyrosine is not uniquely specific for peroxynitrite. The concomitant measurement of nitrotyrosine and dityrosine should provide a better indication [34], albeit indirect measurement of ONOO^−^, which has extremely a short biological half-life. In addition to its short biological half-life, the rate of decomposition of ONOO^−^ has been shown to accelerate in the presence of bicarbonate [35], [36], this may attenuate the rise and preclude the detection of nitrotyrosine level in SB-treated animals. Recently, it has also been shown that both the content of nitric oxide metabolites and expressions of constitutional and inducible nitric oxide synthases were decreased in the cortex of rat kidney after SB loading [37]. Therefore, the observation of lack of changes in cortical nitrotyrosine level in SB-treated piglets could be due to a combination of technical insensitivity and the decrease in bioavailability of endogenous nitric oxide.

Others have reported that the peroxidase activity of SOD can be enhanced dramatically in the presence of SB [38], [39]. The reduction of cortical H_2_O_2_ accumulation after SB administration could alternatively be due to an increase in peroxidation. Bonini et al [40] have demonstrated that peroxymonocarbonate, the product of the spontaneous reaction of bicarbonate with H_2_O_2_, is an intermediate in the SOD peroxidase and is the precursor of carbonate radical anions. Carbonate radical has recently been proposed to be a key mediator of various oxidative damages [41], [42], [43]. The reaction of nitrogen dioxide and carbonate radical on carbohydrates and protein could form a number of nitration and nitrosation products in the endothelial cell of vessels during reperfusion injury [42]. Zhang et al [43] showed that carbonate radicals increased the cytotoxicity of motor neurons in amyotrophic lateral sclerosis. Similarly, increased DNA damage has been reported by the addition of bicarbonate to SOD-H_2_O_2_ system [44]. Since the by-products of SOD peroxidation can cause detrimental effects on oxidative injury, further studies are needed to examine the fate of ROS such as superoxide and ONOO^−^ anions when there is an apparent reduction in cerebral cortical H_2_O_2_ accumulation during hypoxia-reoxygenation by SB.

A clear limitation of our present study is that our relatively short time course (4 h after reoxygenation) will not be able to account for long-term effects of SB on neurodevelopmental outcome. Because of technical difficulty, we were not able to have direct measurement of transient enzymatic changes in the brain during SB infusion. Some of our conclusions are generated based on changes in plasma level and further study needs to be carried out to verify this possibility. The evaluation of differential activities of extracellular, cytosolic and mitochondrial types of SOD will further our understanding of the effect of SB on H_2_O_2_ accumulation. Furthermore, the determination of nitrated proteins (nitrosative modification of proteins) could be very valuable for assessing oxidative stress and nitrosative aggression in tissue. Since it has shown previously that the effects of SB on cerebral hemodynamics and oxygenation in preterm infants depend on the infusion rate, whether different infusion rate would affect the outcome of the present study remains to be investigated.

In summary, the administration of SB rapidly improves the metabolic acidosis, but not hemodynamic recovery, in the resuscitation of asphyxiated newborn piglets. Apparently, it also reduces cortical H_2_O_2_ accumulation without significant effects on oxidative stress markers in the brain probably by the inhibition of dismutation activity of SOD.
